# The impact of PI3K inhibitors on breast cancer cell and its tumor microenvironment

**DOI:** 10.7717/peerj.5092

**Published:** 2018-06-19

**Authors:** Hanjiao Qin, Linlin Liu, Shu Sun, Dan Zhang, Jiyao Sheng, Bingjin Li, Wei Yang

**Affiliations:** 1 Department of Radiotherapy, The Second Hospital of Jilin University, Changchun, Jilin, China; 2 Affiliated Hospital of Changchun University Of Traditional Chinese Medicine, Changchun, Jilin, China; 3 Department of Hepatobiliary and Pancreatic Surgery, The Second Hospital of Jilin University, Changchun, Jilin, China; 4 Jilin Provincial Key Laboratory on Molecular and Chemical Genetic, The Second Hospital of Jilin University, Changchun, Jilin, China

**Keywords:** Breast cancer, PI3K, Pathway inhibitors, Tumor microenvironment, Combination therapy

## Abstract

The phosphoinositide 3-kinase (PI3K) pathway shows frequent aberrant alterations and pathological activation in breast cancer cells. While PI3K inhibitors have not achieved expectant therapeutic efficacy in clinical trials, and several studies provide promising combination strategies to substantially maximize therapeutic outcomes. Besides its direct impact on regulating cancer cells survival, PI3K inhibitors are also demonstrated to have an immunomodulatory impact based on the tumor microenvironment. Inhibition of the leukocyte-enriched PI3K isoforms may break immune tolerance and restore cytotoxic T cell activity by reprogramming the tumor microenvironment. In addition, PI3K inhibitors have pleiotropic effects on tumor angiogenesis and even induce tumor vascular normalization. In this review, we discuss the mechanism of PI3K inhibitor suppression of breast cancer cells and modulation of the tumor microenvironment in order to provide further thoughts for breast cancer treatment.

## Introduction

Breast cancer is the most commonly diagnosed cancer and the leading cause of cancer-related death among women worldwide (Polyak & Metzger Filho, 2012). Despite advances in prevention and in therapeutic measures over the last decades, more than 250,000 new cases of invasive breast cancer and 40,610 breast cancer deaths were estimated in the United States in 2017 (DeSantis et al., 2017). The pathogenesis of breast cancer is associated with pathologic activation of several key signaling pathways, especially the phosphoinositide 3-kinase (PI3K) pathway (Mayer & Arteaga, 2016; Paplomata & O’Regan, 2014; Yang, Polley & Lipkowitz, 2016).

Phosphoinositide 3-kinases are a family of lipid kinases that respond to nutrition, hormones and other environmental cues, and integrate extracellular stimuli into intracellular signals that regulate many biological functions, including cell proliferation, survival, differentiation, metabolism and migration (Thorpe, Yuzugullu & Zhao, 2015). PI3Ks are divided into three classes based on structural and enzyme-kinetic differences: class I, class II and class III. The class I PI3Ks, which comprise PI3Kα, PI3Kβ, PI3Kγ and PI3Kδ, are abnormally activated in breast cancer (Paplomata & O’Regan, 2014; Yang, Polley & Lipkowitz, 2016). Studies have revealed frequent somatic mutations in genes in this pathway, including PIK3CA, PIK3R1, phosphatase and tensin homolog (PTEN) and AKT1, which result in increased PI3K activity or loss of the PTEN functionality (Mayer & Arteaga, 2016; Thorpe, Yuzugullu & Zhao, 2015). Increased activity of the PI3K pathway has been linked with breast cancer tumorigenesis, drug resistance and clinical outcome. Pharmacological efforts have been to target the PI3K pathway in breast cancer, and related experience has been gained from clinical trials.

Recent studies have also revealed the important roles of PI3K inhibition in the tumor microenvironment. The tumor microenvironment enables and supports neoplastic cells to acquire adaptive benefits from the surrounding environment, including the infiltrating tumor-associated immune cells, vasculature and fibroblasts (De Palma, Biziato & Petrova, 2017; Maley et al., 2017). Inhibition of PI3Kδ has not only shown significant therapeutic efficacy in leukemias, but also selectively blocks regulatory T cell-mediated immune tolerance to improve tumor immunotherapy in solid cancer (Ahmad et al., 2017; Ali et al., 2014). Furthermore, several reports showed that PI3Kγ in myeloid cells can reshape the tumor microenvironment between immune suppression and immune stimulation, and even overcome resistance to checkpoint blockade therapy in orthotopic breast cancer mouse models (De Henau et al., 2016; Kaneda et al., 2016b). Other studies have revealed an important role for PI3Kα in regulating tumor angiogenesis in the tumor microenvironment and emerging research has identified a relationship between the PI3K pathway and stromal fibroblasts (Kim et al., 2017; Soler et al., 2013; Trimboli et al., 2009).

Here, we first summarize the PI3K pathway alterations associated with the pathogenesis of breast cancer and the lessons about PI3K pathway inhibitors learned from clinical trails, with rational combination strategies in breast cancer treatment. We next discuss the indirect role of PI3K inhibition on modulating immune cells, angiogenesis and stromal fibroblasts in the tumor microenvironment to influence cancer progression and metastasis.

## Survey Methodology

PubMed was mainly used to search for related articles published using the keyword “breast cancer” “PI3K” “pathway inhibitors” “tumor microenvironment” and “combination therapy”. Then, screened articles were used as references for this review. Additional keywords, such as “cancer,” and “immunotherapy” were also used.

## Pi3k Pathway Signaling and Genetic Alterations in Breast Cancer

In mammals, class I PI3Ks are divided into IA and IB based on different regulation modes. Class IA PI3Ks are activated by receptor tyrosine kinases (RTKs), while class IB are activated by G-protein coupled receptors. PI3Ks function as heterodimeric lipid kinases that consist of a regulatory subunit and a catalytic subunit. In class IA PI3Ks, the p110α, p110β and p110δ catalytic subunits are encoded by PIK3CA, PIK3CB and PIK3CD, respectively, and the p85α, p85β and p55γ regulatory subunits are encoded by PIK3R1, PIK3R2 and PIK3R3 (Thorpe, Yuzugullu & Zhao, 2015). Class IB PI3Ks are heterodimers of a p110γ catalytic subunit, encoded by PIK3CG, together with one of two related regulatory subunits: p101 encoded by PIK3CR5 or p87 encoded by PIK3R6. The p110α and p110β catalytic subunits are ubiquitously expressed in all cell types, whereas p110δ and p110γ expressions are restricted to leukocytes (Vanhaesebroeck et al., 2010).

**Figure 1:**
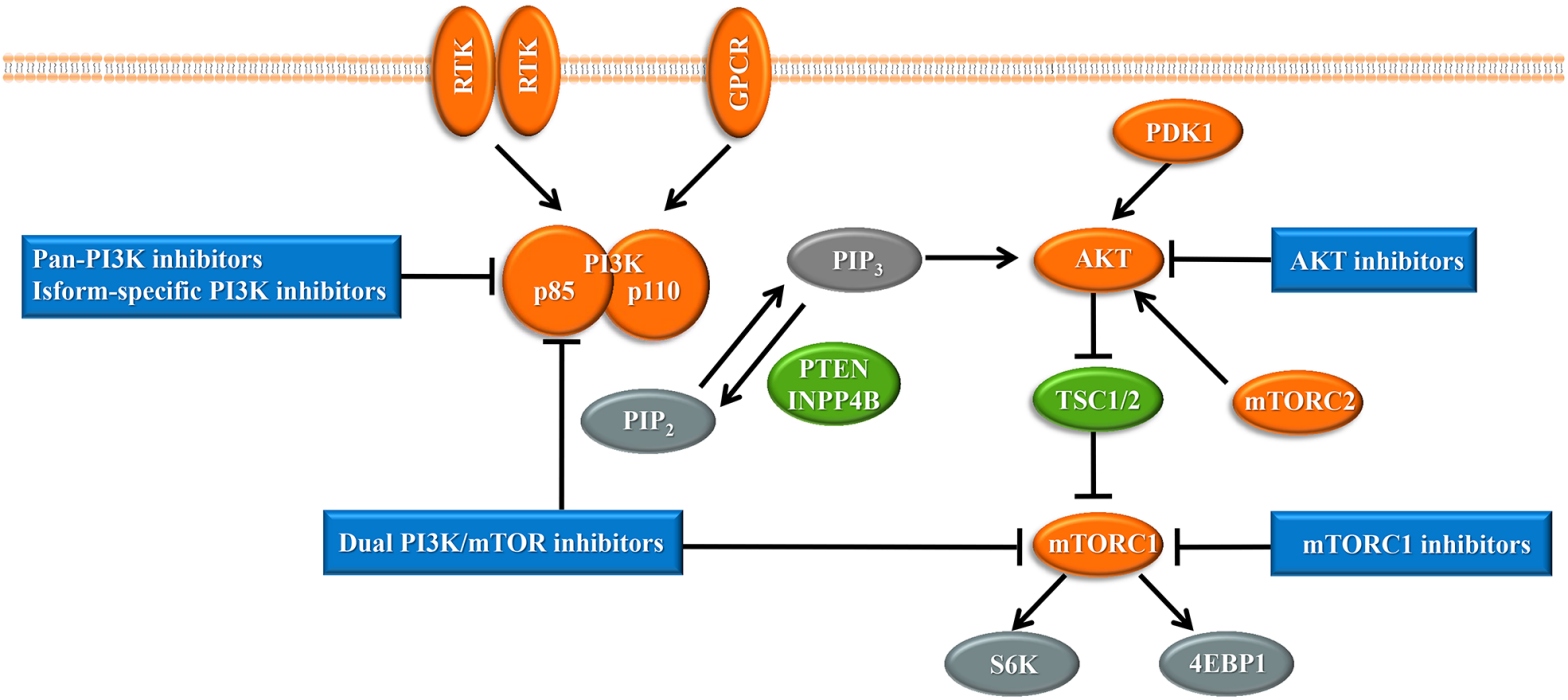
The phosphoinositide 3-kinase pathway and inhibitors of the pathway in cancer. Tumor promoters and suppressors are labeled in orange and green, respectively. Study sites: RTK, receptor tyrosine kinase; GPCR, G-protein-coupled receptor; PI3K, phosphatidylinositol 3-kinase; PIP2, phosphatidylinositol 4,5-bisphosphate; PIP3, phosphatidylinositol 3,4,5-trisphosphate; PDK1, phosphoinositide dependent kinase-1; PTEN, phosphatase and tensin homolog; INPP4B: inositol polyphosphate 4-phosphatase type II; mTORC, mammalian target of rapamycin complex; SK6, S6 kinase; 4EBP1, 4E-binding protein 1.

Upon the presence of activating signals, class I PI3Ks are recruited to the cell membrane, and the catalytic subunit is liberated by the regulatory subunit to phosphorylate phosphatidylinositol 4,5-bisphosphate (PIP2), generating phosphatidylinositol 3,4,5-trisphosphate (PIP3) (Vanhaesebroeck et al., 2010) (Fig. 1). PIP3 acts an important second messenger that coordinates AKT localization to the plasma membrane through specific lipid-binding domains; at the plasma membrane, AKT is then phosphorylated by phosphoinositide dependent protein kinase-1 (PDK1) (Badve & Nakshatri, 2013). AKT functions as the central mediator in the PI3K pathway and activates the downstream signaling pathways, which have a critical influence on the cell cycle and protein synthesis (Yang, Polley & Lipkowitz, 2016). Mammalian target of rapamycin (mTOR) is a serine/threonine protein kinase that comprises structurally related, but functionally distinct multi-component kinase complexes, mTOR complex 1 (mTORC1) and mTOR complex 2 (mTORC2) (Zoncu, Efeyan & Sabatini, 2011). mTORC2 regulates AKT phosphorylation at serine 473 to fully activate AKT (Zoncu, Efeyan & Sabatini, 2011). Activated AKT phosphorylates and inhibits tuberous sclerosis complex 1 and 2 (TSC1/2), which ultimately accumulate and activate mTORC1. mTORC1 is a target of rapamycin and rapamycin analogs and affects cell growth and metabolism by activating 40S ribosomal protein S6 kinase (S6K) and eukaryotic initiation factor 4E binding protein (4EBP1) (Holz, 2012). PTEN negatively regulates the PI3K pathway by dephosphorylating PIP3 into PIP2 (Song, Salmena & Pandolfi, 2012). Inositol polyphosphate 4-phosphatase type II (INPP4B) also functions as a negative regulator by removing phosphatase from PIP3, which is increasingly identified in recent research (Woolley, Dzneladze & Salmena, 2015).

The PI3K pathway is the most frequently mutated in breast cancer through different mechanisms, including (i) increasing PI3K activity by mutation and/or amplification of PIK3CA, PIK3CB, or PIK3R1, (ii) overexpression of activating signals, such as human epidermal growth factor receptor 2 (HER2), epidermal growth factor receptor (EGFR) or insulin-like growth factor 1 receptor signaling, (iii) overexpression of downstream effectors AKT1, AKT2, or PDK1, or (iv) loss of negative regulators such as PTEN and INPP4B (Miller et al., 2011). Constitutive activation of the PI3K pathway induces cellular transformation, and even tumor formation and progression. Among the various mutations of the PI3K pathway, mutation of PIK3CA and loss of the PTEN negative regulator are the most frequently occurring inactivating mechanisms of this pathway in breast cancer. PIK3CA, which encodes the p110α catalytic subunit, is the most common genetic alteration of the AKT pathway in breast cancer, and was first demonstrated as a highly oncogenic gene in 2004 (Liu et al., 2011; Samuels et al., 2004). PIK3CA mutations mostly occur at two hotspot clusters within the helical domain in exon 9 and the kinase domain in exon 20 (Samuels et al., 2004). The H1047R activating mutation in the kinase domain increases the retention of PI3K at the cell membrane, while E542K and E545K mutations in the helical domain enhance catalytic activity and facilitate interactions with substrates (Mayer & Arteaga, 2016). The PTEN tumor suppressor is significantly reduced in about 25% of breast cancer (She et al., 2016). Triple-negative breast cancer, characterized by lack of HER2 and hormone receptor expression, has a particularly poor prognosis, and this subtype is associated with a 30% rate of PTEN alteration (She et al., 2016). Growing evidence demonstrates a central role for PTEN in cancer stem cell homeostasis. Deletion of PTEN leads to expansion of leukemia-initiating cells and contributes to leukemia (Zhang et al., 2006). In addition, PTEN also has an important impact on the tumor microenvironment and regulates metabolic requirements (Song, Salmena & Pandolfi, 2012).

## Inhibitors of the Pi3k Pathway and Combination Therapeutic Strategies in Breast Cancer

With the central role of the PI3K pathway in breast cancer, inhibiting excessive activation of this pathway is a promising anti-cancer treatment strategy. The selective mTORC1 inhibitor everolimus, the rapamycin analogues, is already United States FDA-approved for the treatment of hormone receptor-positive, HER2-negative breast cancer (Bauer, Patel & Infante, 2015). Enormous efforts have focused on the development of drugs targeting other molecules in the PI3K pathway, such as pan-PI3K inhibitors, isoform-specific PI3K inhibitors, AKT inhibitors, and dual PI3K/mTOR inhibitors, and several are already undergoing clinical trials (Fig. 1). Despite the established connection between the PI3K pathway and the progression of breast cancer, PI3K inhibitors have not achieved expectant therapeutic efficacy in clinical trials (Rodon et al., 2013). Compared with drugs targeting other oncogenic kinases, PI3K inhibitors have shown only modest effectiveness on patients to date (Okkenhaug, Graupera & Vanhaesebroeck, 2016). Furthermore, breast cancer cells easily acquire resistance to these PI3K pathway inhibitors (Toska & Baselga, 2016). These observations may be because cancer cells escape from single-target agents by increasing the transcription of upstream RTKs (Chandarlapaty et al., 2011). Several clinical trials have found that treatment of breast cancer with PI3K inhibitors results in upregulated activity of the estrogen pathway (Miller, Balko & Arteaga, 2011). Other studies indicated that PI3K inhibition increased DNA damage and induced drug resistance in a genetically engineered mouse model of breast cancer (Juvekar et al., 2012). These data suggest that rational combination of PI3K inhibitors with other therapeutics may overcome resistance to maximize therapeutic outcomes (Table 1).

**Table 1:** Schematic representation of rational combination PI3K inhibitors with other therapeutics in breast cancer.

Combination therapeutic strategies	Applied drugs	Type of study	Tpye of breast cancer	Reference
PI3K and RTK inhibitors	p110-selective inhibitor (BYL719) & HER3-neutralizing antibody (LJM716)	in vitro and in vivo	HER2-posotive breast cancer	Garrett et al. (2013)
	PI3K inhibitor (GDC-0941) & dual EGFR and HER3 inhibitor (MEHD7945A)	in vitro and in vivo	Triple-negative breast cancer	Tao et al. (2014)
	AKT inhibitor (MK-2206) & HER2 inhibitor (trastuzumab)	Phase I trial	HER2-posotive breast cancer	Hudis et al. (2013)
	PI3K inhibitor (BKM120/buparlisib) & HER2 inhibitor (trastuzumab)	Phase IB trial	HER2-positive advanced or metastatic breast cancer	Saura et al. (2014)
	PI3K inhibitor (buparlisib) & HER2 inhibitor (lapatinib)	Phase IB trial	HER2-positive advanced breast cancer	Guerin et al. (2017)
	PI3K inhibitor (BKM120/buparlisib) & HER2 inhibitor (trastuzumab)	Phase II trial	HER2-positive locally advanced or metastatic breast cancer	Pistilli et al. (2018)
PI3K inhibitors and endocrine therapy	PI3K inhibitor (pictilisib) & ER antagonist (fulvestrant)	Randomized double-blind phase II trial	ER-positive, HER2-negative metastatic breast cancer	Krop et al. (2016)
	PI3K inhibitor (pictilisib) & aromatase inhibitor (anastrozole)	Phase II randomized preoperative window-of-opportunity study	ER-positive breast cancer	Schmid et al. (2016)
	PI3K inhibitor (buparlisib) & ER antagonist (fulvestrant)	Randomized double-blind placebo-controlled phase III trial	ER-positive HER2-negative advanced breast cancer	Baselga et al. (2017)
	PI3K inhibitor (buparlisib) & ER antagonist (fulvestrant)	Randomized double-blind placebo-controlled phase III trial	ER-positive HER2-negative advanced breast cancer progressing on or after mTOR inhibition	Di Leo et al. (2018)
	p110-selective inhibitor & ER antagonist (fulvestrant)	Phase III trial	HR-positive or OR-positive HER2-negative advanced breast cancer progressing on or after mTOR aromatase inhibitor	ongoing trial
PI3K and PARR inhibitors	PI3K inhibitor (NVP-BKM120) & PARP inhibitor (Olaparib)	in vivo	BRCA1-related breast cancer	Juvekar et al. (2012)
	PI3K inhibitor (BKM120) & PARP inhibitor (Olaparib)	in vitro and in vivo	BRCA-proficient triple-negative breast cancer	Ibrahim et al. (2012)
	Dual PI3K and mTOR inhibitor (GDC-0980) & PARP inhibitor (ABT888)	in vitro and in vivo	Triple negative breast cancer	De et al. (2014)
	PI3K inhibitor (BKM120) & PARP inhibitor (Olaparib)	Phase I trial	High-grade breast cancer	Matulonis et al. (2017)
PI3K inhibitors and immune checkpoint targeting agents	PI3K-inhibiting supramolecule & anti-PD-1 antibody	in vivo	4T1 breast cancer	Kulkarni et al. (2016)
	PI3Kγ inhibitor (TG100-115) & anti-PD-1 antibody (clone RPM1-14)/anti-CTLA4 antibody (clone 9H10)	in vivo	4T1 breast cancer	De Henau et al. (2016)
	PI3K inhibitor (BKM120) & anti-PD-1 antibody	in vivo	4T1/PyMT breast cancer patient-derived triple negative breast cancer	Sai et al. (2017)

**Notes:**

PI3K, phosphatidylinositol 3-kinase; RTK, receptor tyrosine kinase; HER3, epidermal growth factor receptor 3; EGFR, epidermal growth factor receptor; ER, estrogen receptor; PARP, poly-ADP-ribosylation; mTOR, Mammalian target of rapamycin; HER2, epidermal growth factor receptor 2.

### PI3K and RTK Inhibitors

Several studies have shown that inhibition of the PI3K pathway induces the expression and phosphorylation of multiple RTKs, such as HER2, via the forkhead box O-regulated transcriptional process in breast cancer cells (Chandarlapaty et al., 2011; Garrett et al., 2011; O’Reilly et al., 2006; Tao et al., 2014). Especially in HER2-positive breast cancer, the upregulation of HER3 was detected in response to PI3K inhibition (Chakrabarty et al., 2012; Chandarlapaty et al., 2011; Garrett et al., 2011, 2013; Tao et al., 2014). HER3 is a kinase-defective member of the HER kinase family, and overexpressed HER2 can dimerize with HER3 to form HER2-HER3 heterodimers, which bind to and activate PI3K (Chandarlapaty et al., 2011). With sustained inhibition of mTOR, breast cancer cells can take advantage of upstream RTK feedback signaling to acquire resistance to drugs. Preclinical studies have shown that a combination of HER3-neutralizing antibody and a p110α-selective inhibitor markedly reduced the growth of breast cancer xenografts (Garrett et al., 2013). Another study showed the effectiveness of a combination of PI3K inhibitor and a dual EGFR and HER3 inhibitor to bypass this resistance mechanism (Tao et al., 2014). In addition, more evidence has indicated PIK3CA mutations mediating resistance to HER2 targeted agent in patients with HER2-positive solid cancer (Berns et al., 2007; Black et al., 2015; Rimawi et al., 2018). The wild-type breast cancer cell lines transfected with constitutively oncogenic PIK3CA mutations showed almost insensitive toward trastuzumab, suggesting the major role of PIK3CA in the development of resistance to trastuzumab (Berns et al., 2007). Importantly, it is demonstrated that PIK3CA mutations are associated with lower pathological complete response (pCR) rates to HER2 targeted therapy in primary HER2-positive breast cancer (Loibl et al., 2014, 2016; Majewski et al., 2015; Rimawi et al., 2018). In a pooled analysis of five prospective clinical trials evaluating lapatinib and trastuzumab, the pCR rate for PIK3CA mutation versus wild-type was 16.7% versus 39.1% (*P* < 0.001) (Loibl et al., 2016). Taken together, these studies suggest that simultaneous inhibition of PI3K and RTK is an effective therapeutic strategy to enhance drug efficacy.

A phase I trail was conducted to evaluate the safety of combination AKT inhibitor MK-2206 with trastuzumab, results indicating its safety and clinical activity (Hudis et al., 2013). The other two phase IB studies aimed to determine tolerability and maximum tolerated dose for buparlisib in combination with HER2 targeted drugs in HER2-positive, trastuzumab-resistant, advanced breast cancer (Guerin et al., 2017; Saura et al., 2014). In this patient population, the combination was well tolerated and preliminary evidence of antitumor activity were observed. Based on the recommended phase II dose of burparlisib as 100 mg/day in combination with 2 mg/kg weekly tratuzumab from phase IB, 50 patients with HER2+ locally advanced breast cancer resistant to trastuzumab-based treatment were treated with this therapeutic regimen (Pistilli et al., 2018). The overall response rate was only 10% and the primary endpoint was not met, demonstrating limited efficacy in this phase II study. Therefore, more evidence needs to be gained from ongoing clinical trials.

### PI3K inhibitors and endocrine therapy

The crosstalk between PI3K activity and estrogen receptor (ER) signaling has been identified, as the PI3K pathway regulates the ER pathway both directly and indirectly (Miller, Balko & Arteaga, 2011). AKT phosphorylates ER at Ser167 to increase estrogen-induced ER transcriptional activity, and PI3K indirectly activates ER transcription by means of c-Jun complexes interacting with c-Foc to form AP-1 complexes (Bosch et al., 2015; Petz et al., 2002). In addition, emerging evidence has shown that estrogen signaling also has an impact on the PI3K pathway, as estrogen stimulation activates intracellular kinase pathways, including PI3K, IGF-1R, and EGFR (Miller et al., 2009; Song et al., 2006). Preclinical research indicated that suppression of PI3K signaling in an ER/PIK3CAmut model induced activation of ER-dependent transcription, including the ER promoter and genes with ER-binding sites in the coding sequence (Bosch et al., 2015). Interestingly, another study showed that reduced PTEN levels led to endocrine resistance in ER-positive breast cancer, which can be overcome by combination endocrine therapy with a PI3K inhibitor (Fu et al., 2014). Therefore, combined inhibition of PI3K and ER may be an effective strategy for breast cancer, and several clinical trials are undergoing.

A randomized double-blind phase II trial in ER-positive, HER2-negative metastatic breast cancer that had been resistant to treatment with aromatase inhibitor indicated that therapy efficiency did not benefit from the addition of the pan-class I PI3K inhibitor pictilisib (Genentech; GDC-0941) to fulvestrant (an ER antagonist) (Krop et al., 2016). The reason underlying the lack of significantly improved progression-free survival was because of toxicity limitations with pictilisib. Another phase II randomized preoperative window-of-opportunity study in postmenopausal women with newly diagnosed, operable ER-positive breast cancer showed that adding pictilisib to anastrozole (an aromatase inhibitor) significantly enhanced suppression of breast cancer cell proliferation compared with anastrozole alone (Ki 67 staining; 83.8% vs. 66.0%) (Schmid et al., 2016). Further, the sensitivity to pictilisib was independent of PIK3CA mutations and the combination treatment showed a remarkable anti-proliferation effect in luminal B primary breast cancer (Ki 67 staining; 37%). In addition, a randomized double-blind placebo-controlled phase III trial in postmenopausal, HR-positive, HER2-nagative, advanced breast cancer was performed to assess buparlisib plus fulvestrant (Baselga et al., 2017). The results of this BELLE-2 trail indicated that pan-PI3K inhibitor with endocrine therapy was clinically meaningful benefit in the total patient population (median progression-free survival: 6.9 months vs. 5.0 months), while serious adverse were reported as high as 23% in the buparlisib group compared with 16% in the placebo group. In consideration of PI3K inhibitor down-regulating the phosphorylation of AKT following mTOR inhibition, the phase III, randomized, placebo-controlled BELLE-3 trail was designed to test buparlisib combination with fulvestrant in patients who relapsed on or after endocrine therapy and mTOR inhibitors (Di Leo et al., 2018). However, the safety profile of the therapeutic regimen was not recommended to further development. Interestingly, preclinical studies have suggested that oncogenic PIK3CA driven the development of breast cancer (Liu et al., 2011; Utermark et al., 2012), and the PI3Kα-selective inhibitors shown more effective and more tolerable toxicity profile in clinical trails (Juric et al., 2018; Mayer et al., 2017). Phase III trials investigating α-selective PI3K inhibitors combined with ER antagonist in the endocrine resistant setting are currently ongoing, including SOLAR-1 and SANDPIPER.

### PI3K and poly-ADP-ribose polymerase inhibitors

Two studies provided the first evidence that a PI3K inhibitor induces impairment of DNA homologous recombination in BRCA-proficient triple-negative breast cancer, with an increase in poly-ADP-ribosylation and downregulation of BRCA1/2 (Condorelli & Andre, 2017; Ibrahim et al., 2012; Juvekar et al., 2012; Rehman, Lord & Ashworth, 2012). The authors also showed that adding the poly-ADP-ribose polymerase (PARP) inhibitor olaparib to PI3K inhibitors markedly attenuated tumor activity in triple negative breast cancer with wild-type BRCA1. Pradip et al., conducted a study using BRCA-competent breast cancer-bearing mouse models and showed that GDC-0980 (a dual PI3K-mTOR inhibitor) enhanced the antitumor effects of the PARP inhibitor ABT888 plus carboplatin by inhibition of tumor cell proliferation and tumor-induced angiogenesis; however, combination with GDC-0941, a pan-PI3K inhibitor, failed to suppress tumor growth in MDA-MB231 breast cancer cells (De et al., 2014). These results indicate that co-administration of PI3K inhibitor and PARP inhibitor may a rational therapeutic strategy.

Based on these preclinical studies in animal models, a phase I clinical trial was performed on the combination of the PI3K inhibitor BKM120 and the PARP inhibitor olaparib to test side effects and the maximum tolerated dose (Matulonis et al., 2017). Clinical benefit of the combination treatment was observed in patients with germline BRCA mutation or germline wild-type BRCA, but the dose of BKM120 is limited by the co-administered drugs because of its toxicity. With further clinical study of PI3K inhibitors, other PI3K inhibitors might be more suitable for combination therapy. Randomized phase II studies will be conducted to further confirm the efficacy of the PI3K/PARP inhibitor combination.

### Combination therapy in breast cancer: further evidence to explore

Preclinical and clinical studies have provided several potential combination PI3K inhibitors and other therapeutics, but more evidence is needed to clarify additional questions. Whether targeted kinase inhibitors are dependent on the PI3K molecular alteration should be examined. Interestingly, several studies revealed a key effect of oncogenic PIK3CA on mammary cell fate, which activates a multipotent genetic program at the early stage of tumor initiation and controls tumor heterogeneity (Koren et al., 2015; Van Keymeulen et al., 2015). Whether inhibition of PI3K has an impact on blocking PIK3CA-induced initiation of malignancy still needs to be verified by more research. Additional studies should also focus on identifying the patients who benefit from the combination treatment.

## Indirect Role of Pi3k Pathway Inhibition in Breast Cancer Based on the Tumor Microenvironment

The driving force of tumor initiation is the induction of mutations in cancer cells by genome instability (Hanahan & Coussens, 2012). Yet these neoplastic cells cannot complete the transforming process alone (Hanahan & Coussens, 2012), so they recruit bone marrow-derived stromal stem cells and progenitor cells to form the tumor-associated stroma in solid cancers, which is increasingly known as the tumor microenvironment (Hanahan & Weinberg, 2011). The recruited immune cells that have been remodeled by cancer cells progressively create a succession of changes to support multistep transformation into malignancy (McAllister & Weinberg, 2014). With the accepted importance of tumor microenvironment, it is considered to represent a promising therapeutic direction (Ivey et al., 2016). There are several potential therapeutic targets in the tumor microenvironment, such as tumor-promoting inflammatory cells, cancer-associated fibroblasts, and vascular endothelial cells (Hanahan & Coussens, 2012; Ivey et al., 2016). While the direct effects of PI3K inhibition on breast cancer cells are significant, overwhelming research suggests that PI3K inhibition indirectly affects breast cancer cell by targeting the tumor microenvironment (Ali et al., 2014; Peng et al., 2016a; Sai et al., 2017). The expressions of PI3Kδ and PI3Kγ are restricted to leukocytes (Vanhaesebroeck et al., 2010), and recent animal experiments indicated that pharmacological inhibition or genetic ablation of PI3Kδ and PI3Kγ may modulate the immune response to enhance antitumor efficacy in breast cancer (Ali et al., 2014; De Henau et al., 2016). Further, PI3K inhibition directly interferes with tumor-associated angiogenesis by endothelial cells or by indirect inhibition of angiogenesis-promoting myeloid cells (Rivera et al., 2015; Soler et al., 2016). We next focus on how PI3K inhibitors impact the tumor microenvironment in breast cancer (Table 2).

**Table 2:** Schematic representation of PI3K isoform-specific roles in breast cancer based on the tumor microenvironment.

TME	PI3K isoform-specific inhibitor or PI3K negative regulator	Modulate TME component	Effects on breast cancer	Reference
Stromal fibroblasts	PTEN	Fibroblasts	Suppress mammary epithelial tumors; remodel the extracellular matrix, the innate immune cell infiltration and angiogenesis.	Trimboli et al. (2009)
	PTEN	Fibroblasts	Suppress mammary epithelial tumors; control the interactions between tumor and stromal.	Wallace et al. (2011)
	PTEN	Fibroblasts	Inhibit expansion of mammary epithelial stem cells; regulate mammary ductal elongation and alveolar side-branching.	Sizemore et al. (2017)
Tumor angiogenesis	PI3Kγ inhibitor	MDSCs, TAMs	Suppress tumor angiogenesis by alter the inflammatory environment.	Schmid et al. (2011)
	PI3K inhibitor	MDSCs	Generate an enduring angiostatic and Immune-stimulatory environment in which anti-angiogenic therapy remained efficient.	Rivera et al. (2015)
Tumor-infiltrating immune cells	PI3Kδ inhibitor	Tregs, MDSCs	Reduce primary tumor mass and metastasis, with longer survival time; break tumor-induced immune tolerance.	Ali et al. (2014)
	PI3Kγ inhibitor	MDSCs, TAMs	Strongly inhibit spontaneous breast carcinoma; strongly reduce myeloid cell and macrophage recruitment.	Schmid et al. (2011)
	PI3Kγ inhibitor	TAMs	Promote breast cancer regression and extend survival promoting and immunostimulatory transcriptional program.	Kaneda et al. (2016b)
	PI3Kγ inhibitor	MDSCs	Overcome resistance to immune checkpoint blockade therapy in breast cancer.	De Henau et al. (2016)
	PI3Kγ inhibitor	Immune cells	Reduce primary tumor growth, enhance anti-tumor immunity, and heighten susceptibility to immune checkpoint inhibitors.	Sai et al. (2017)

**Notes:**

TME, tumor microenvironment; PI3K, phosphatidylinositol 3-kinase; PTEN, phosphatase and tensin homolog; TAM, tumor-associated macrophage; MDSC, myeloid-derived suppressor cell; Treg, regulatory T lymphocyte.

### Impact of PI3K inhibitors on stromal fibroblasts

The interactions of stromal cells and epithelial cells play a pivotal role in normal mammary development, including in regulating epithelial cell polarity and determining mammary duct formation, and the dysfunction of this interaction results in mammary epithelial cell proliferation and even malignant transformation (Mao et al., 2013; Place, Jin Huh & Polyak, 2011). Fibroblasts, the abundant and critical cell type in the normal mammary stroma, serve as the basic cellular component of connective tissue and maintain the extracellular environment by secreting and remodeling the extracellular matrix (ECM) (Mao et al., 2013; Soysal, Tzankov & Muenst, 2015). Cancer-associated fibroblasts, with higher proliferative and other cellular activities, produce increasing levels of various growth factors, cytokines, and ECM-degrading proteases, and substantial data support a role for these cancer-associated fibroblasts in breast cancer progression (Aboussekhra, 2011; Orimo et al., 2005; Tyan et al., 2011). Orimo and colleagues showed that cancer-associated fibroblasts extracted from human breast cancer significantly promote the growth of breast cancer cells and increase angiogenesis by recruiting endothelial progenitor cells (Orimo et al., 2005).

Phosphatase and tensin homolog, as a negative regulator of PI3K, has been extensively demonstrated to function in the stromal fibroblasts to suppress mammary epithelial tumors (Trimboli et al., 2009; Wallace et al., 2011). Genetic ablation of PTEN in stromal fibroblasts of mouse mammary glands have been shown to remodel the ECM by increasing the level of the transcription factor Ets2, which directly targets the promoters of MMP9 and CCL3 (Trimboli et al., 2009). As a result, the PTEN stromal-deleted breast cancers exhibit high levels of innate immune cell infiltration and tumor vascular structures (Trimboli et al., 2009). Another recent study further indicated that fibroblast-specific PTEN inactivation results in failure of elongation of mammary ducts and promotes abnormal alveolar side-branching (Sizemore et al., 2017). Importantly, the PTEN-null mice showed an expansion of the mammary epithelial stem cell-enriched basal/myoepithelial population and well-defined activity of the stem cells tested in vitro (Sizemore et al., 2017). In a pancreatic cancer mouse model, the inhibition of PI3Kγ was demonstrated to reduce cancer-associated fibroblast-induced collagen production by regulation of tumor associated macrophages (Kaneda et al., 2016a).

### Pleiotropic impact of PI3K inhibitors on tumor angiogenesis

Tumor vessel formation is critical for persistently supplying nutrients and oxygen for high proliferating cancer cells, and indeed, aberrant tumor vessel structure and function are hallmarks of cancer (Hanahan & Weinberg, 2011). Microvascular density is an independent prognostic indicator in invasive breast cancer (Arora et al., 2002; Nieto et al., 2007; Tsutsui, Kume & Era, 2003), and high microvascular density in breast cancer is associated with tumor invasiveness and metastasis (Ribatti et al., 2016). A variety of tumor microenvironment cells are involved in the formation of tumor-associated neovasculature. Endothelial cells respond to tumor overexpressed angiogenic signals and are activated to form the inner surface of tubular vessel (Potente, Gerhardt & Carmeliet, 2011), and the infiltrating immune cells are recruited to promote tumor angiogenesis by releasing angiogenic factors (Hanahan & Coussens, 2012). Remarkably, PI3K isoforms each function in these cell types, such as PI3Kα in endothelial cells (Soler et al., 2013), PI3Kγ/PI3Kδ in immune cells and PI3Kβ in platelets (Martin et al., 2010; Rivera et al., 2015). It is therefore likely that PI3K inhibition has pleiotropic effects on angiogenesis by different mechanisms.

#### PI3Kα in endothelial cells: the direct vascular impact

A majority of preclinical studies indicate that PI3Kα activity in endothelial cells is essential for vascular development, and the inactivation of the endothelial cell-specific p110α contributes to embryonic lethality due to severe vascular defects (Graupera et al., 2008; Gupta et al., 2007; Lelievre et al., 2005; Soler, Angulo-Urarte & Graupera, 2015). In mice models and human diseases, PIK3CA mutation causes malformations and dysfunction of lymphatic vessels (Boscolo et al., 2015; Gupta et al., 2007; Stanczuk et al., 2015) and venous vessels (Castel et al., 2016; Castillo et al., 2016; Limaye et al., 2015). PI3Kα-selective inhibitors in stromal cells lead to increased vessel density, reduced vascular volume, and altered pericyte coverage, which is summarized as the nonfunctional angiogenesis causing suppression of cancer growth (Soler et al., 2013). In addition, tumor-bearing murine models have shown that inhibition of p110α results in stunted tumor angiogenesis, which may be explained by the interaction between Ras and PI3Kα regulating vascular endothelial growth family signaling as well as the growth-permissive tumor microenvironment (Murillo et al., 2014; Soler et al., 2016).

#### Vascular effect of non-PI3Kα isoforms on cancer

PI3Kγ was demonstrated to play an important role in endothelial progenitor cell functions and neovascularization as early as 2008 (Madeddu et al., 2008). Another study showed that myeloid cell-specific PI3Kγ is required for tumor angiogenesis as well as metastasis by altering the inflammatory environment, with a significant change of VEGFA expression in orthotropic breast cancer mice models (Schmid et al., 2011). Further studies in a mouse model genetically deleted for p110γ or with selective pharmacological inhibition of macrophage-dominant p110γ showed reduced levels of HIF protein and its associated transcriptional target VEGF (Joshi et al., 2014). PI3K isoforms in myeloid cells, both p110γ and p110δ, promote immune suppression and tumor neovascularization during antiangiogenic therapy, and inhibiting PI3K in these cells may improve the effectiveness of antiangiogenic therapy in PyMT mammary cancer models (Rivera et al., 2015). Furthermore, numerous studies have shown that the p110β isoform of PI3K in platelets controls platelet recruitment and activation (Consonni et al., 2012; Martin et al., 2010). In addition to participating in angiogenesis, platelets have a regulatory effect on the vascular remodeling of tortuous vasculature during tumor growth and metastasis (Andrade et al., 2017; Gay & Felding-Habermann, 2011; Yan et al., 2014). A recent study also showed that PI3Kβ-silenced breast cancer cells have reduced lung metastasis by regulating extravasation, which may be associated with disrupting macrophage-induced tumor cell invasion (Khalil et al., 2016).

#### PI3K inhibitors induce tumor vascular normalization

In addition to examining different PI3K isoforms associated with tumor angiogenesis, other research has also focused on the role of PI3K inhibition in altering vascular function (Okkenhaug, Graupera & Vanhaesebroeck, 2016; Soler, Angulo-Urarte & Graupera, 2015). Remarkably, using low doses of PI3K inhibitors may regulate tortuous and immature tumor vessels into the regularity of endothelial cell structure, pericyte coverage and tight junctions, and the persistent modulation of vasculature improves tumor hypoxia and vascular perfusion, with reduced metastatic nodules in distant organs (Fokas et al., 2012; Kim et al., 2017; Qayum et al., 2012; Qayum et al., 2009). Surprisingly, the so-called vascular normalization also enhances delivery of chemotherapy and responsiveness to radiotherapy, suggesting PI3K inhibitors as enhancers in cancer treatment (Fokas et al., 2012; Kim et al., 2017; Qayum et al., 2012). Taken together, these results highlight the importance of PI3K inhibitor-induced tumor vascular alterations and the need to validate practical benefits in clinical trials.

### Impact of PI3K inhibitors on tumor-infiltrating immune cells

Tumor-infiltrating immune cells constitute a major cell population of the breast cancer microenvironment and are reprogrammed by breast cancer cells to support tumor onset and progression by suppressing the antitumor immune response (Jezierska-Drutel, Rosenzweig & Neumann, 2013; Place, Jin Huh & Polyak, 2011; Soysal, Tzankov & Muenst, 2015). Immunosuppression regulatory T cells (Tregs) are involved in immune tolerance to turn into more aggressive and higher grade breast cancer (Bohling & Allison, 2008; Ohara et al., 2009; Plitas et al., 2016), while CD8+ T lymphocytes infiltrating in invasive breast cancer have been considered as a good predictive and prognostic marker (Al-Saleh et al., 2017; Mahmoud et al., 2012). Myeloid-derived suppressor cells play a crucial role in breast cancer malignant individualities and are recruited by tumor cells to produce and secrete pro-tumor cytokines (Chen, Zhang & Massague, 2011; Peng et al., 2016b; Shou et al., 2016). Further, the immunosuppressive effect of these cells is associated with weakening effectiveness of immunotherapy and even resistance to checkpoint blockade therapy in breast cancer (De Henau et al., 2016; Shou et al., 2016). Surprisingly, more and more evidence has suggested that PI3K isoforms in leukocytes have an effect on innate and adaptive immune responses, such as PI3Kδ in regulatory T cells and PI3Kγ in myeloid cells (Ahmad et al., 2017; Ali et al., 2014; De Henau et al., 2016). Given the significance of the immune reaction in breast cancer, we focus on the the immunomodulatory impact of PI3K inhibition on tumor regression and the promising benefit to enhance immunotherapy.

#### PI3Kδ inhibition in regulatory T cells: unleash the anti-tumor immune response

Regulatory T cells as immunosuppressive cells play a role in pro-tumor reactivity in breast cancer (Bailur et al., 2015; Li et al., 2017). The ability of PI3Kδ inhibitors to interfere with Tregs in hematological malignancies is well established, and a preclinical study indicated that genetic deletion or selective pharmacological inactivation of p110δ has a significant effect in solid cancers by dampening the function of Tregs (Ali et al., 2014). Compared with wild-type mice, PI3Kδ kinase-inhibition mice are more resistant to B16 melanoma, with a significantly decreased tumor incidence and almost absent for lymph mode metastasis; similar observations were seen in these mice with 4T1 breast cancer, which showed an even longer survival time (Ali et al., 2014). P110δ inhibition in Tregs has been responsible for the immunomodulatory mechanism by unleashing CD8+ cytotoxic T cells to induce tumor regression (Ali et al., 2014; Patton et al., 2006). PI3Kδ-deletion Tregs also have reduced levels of CD38 expression, which fails to upregulate suppression activity-related surface protein (Patton et al., 2011). In addition, recent research indicated that PI3Kδ inhibition may delay CD8+ T cell terminal differentiation but maintain the memory phenotype to improve proliferation ability and cytokine production; this was confirmed in B16 melanoma mouse xenograft models showing PI3Kδ inhibition improved anti-tumor therapeutic efficiency and prolonged survival (Abu Eid et al., 2017).

The exact impact of PI3Kδ in CD8+ T cells is not clear. Some studies indicate that PI3Kδ controls the magnitude of the effector T cell response during infections (Gracias et al., 2016; Jarmin et al., 2008; Pearce et al., 2015; Putz et al., 2012; Soond et al., 2010). The intact formation of long-term memory T cell activity is preserved independently of p110δ (Pearce et al., 2015), which may be a plausible reason why PI3Kδ kinase-ablation suppressed tumor progression by CD8+ cytotoxic T cells (Abu Eid et al., 2017; Ali et al., 2014). An effective immune response requires the recruitment of T lymphocytes from the bloodstream to the targeted location, and one study showed that PI3Kδ activity in T lymphocytes is necessary for efficient T cell receptor-induced migration and localization (Jarmin et al., 2008). However, the authors also showed that p110δ is not required for antigen-independent T cell transendothelial migration and chemotaxis, with constitutive T cell trafficking not affected by p110δ activity (Jarmin et al., 2008).

#### PI3Kγ inhibition in myeloid cells: switch to immune stimulation

Many studies have demonstrated the relationship between inflammation and cancer (Hanna et al., 2017; Tobias et al., 2017), and PI3Kγ is highly expressed in immune cells of myeloid origin. PI3Kγ-deficient mice treated with dextran sulfate sodium are resistant to colitis-associated cancer with defective activation and infiltration of macrophages and neutrophils, suggesting the significant role of PI3Kγ in regulating the innate immune response (Gonzalez-Garcia et al., 2010). Genetic or pharmacological blockade of p110γ in mice implanted with tumor cells have substantially suppressed tumor inflammation by decreasing myeloid cell invasion into tumors, which significantly prevents tumor growth, angiogenesis and metastasis (Foubert, Kaneda & Varner, 2017; Sai et al., 2017; Schmid et al., 2011; Schmid et al., 2013). Further, the inhibition of PI3K p110γ blocks spontaneous breast cancer progression by reducing myeloid cell trafficking without directly altering tumor cells (Schmid et al., 2011). The mechanism of PI3Kγ controlling myeloid cell recruitment to tumors is dependent on Rap1 α-mediated activating the single integrin α4β1 (Foubert, Kaneda & Varner, 2017; Schmid et al., 2011, 2013). Plastic macrophages in neoplasm are reshaped by the tumor microenvironment to display the immunosuppressive phenotype, which produces anti-inflammatory cytokines to inhibit cytotoxic T cells (Lewis, Harney & Pollard, 2016; Qian & Pollard, 2010). Unexpectedly, macrophage PI3Kγ controls a critical switch between immune suppression and stimulation during inflammation and cancer, which induces a transcriptional program by inhibiting NFκB activation but stimulating C/EBPβ activation (Kaneda et al., 2016b). In contrast, selective blockade of PI3Kγ polarizes tumor-associated macrophages to restore CD8+ T cell-mediated cytotoxic activity, thus promoting tumor regression and extending survival time in various mouse cancer models (De Henau et al., 2016; Foubert, Kaneda & Varner, 2017; Kaneda et al., 2016a, 2016b). In addition to the role of PI3Kγ in regulating myeloid cells, the p110δ isoform also has an impact on myeloid-derived suppressor cells and is associated with cancer progression and antitumor effects in 4T1 breast tumor-bearing mouse models (Ali et al., 2014).

#### PI3K inhibition in immune cells: enhance cancer immunotherapy

Tumor-induced immune suppression poses an obstacle to efficacious anti-tumor immunotherapy, especially immune checkpoint blockade therapy. As previous research has shown, the immunomodulatory impact of PI3K inhibition on immune cells of the tumor microenvironment has the potential to enhance immunotherapy. PI3Kγ inhibition in myeloid cells facilitated susceptibility to anti-PD1 responses in triple negative breast cancer animal models (Sai et al., 2017), and even overcame resistance to immune checkpoint inhibitors (De Henau et al., 2016; Kaneda et al., 2016b). Similarly, a PI3Kδ-specific inhibitor increased numbers of tumor vaccine-induced CD8+ T cells and promoted antitumor efficiency by reducing Treg function (Ahmad et al., 2017). The likely mechanism involves PI3K inhibition targeting immune suppressor cells to reeducate the tumor immune microenvironment and restore cytotoxic T cell activity (Ahmad et al., 2017; De Henau et al., 2016; Kaneda et al., 2016b; Sai et al., 2017). In addition, preclinical and clinical studies show that loss of PTEN resulted in inhibition of T-cell infiltration into tumors and T cell-induced tumor killing, eventually promoting resistance to T cell-mediated immunotherapy in melanoma (Peng et al., 2016a). This suggests that treatment with a selective PI3K inhibitor is a strategy to increase efficacy of immunotherapy.

## Conclusions

In this review, we focused on the impact of the PI3K family on breast cancer initiation and progression, including direct and indirect effects on cancer cells based on the tumor microenvironment. PI3K inhibition in breast cancer cells, as a therapeutic target, has shown relatively moderate effectiveness as a monotherapy in clinical trials. Several combinations of PI3K inhibitors with other targeted drugs significantly maximized therapeutic outcomes, and several clinical trials are undergoing to confirm these strategies. With the established importance of the tumor microenvironment in solid cancer, emerging evidence highlights the potential of PI3K isoforms in regulating constituents of the tumor microenvironment, such as tumor-promoting immune cells, cancer-associated fibroblasts, and vascular endothelial cells. In addition to the direct impact of PI3K inhibition on breast cancer cell, inhibition of PI3K isoforms may restore the anti-tumor immune response and improve responsiveness to checkpoint blockade treatment, which may be a promising immunotherapy target for breast cancers. These therapeutic concepts should be pursued with clinical studies to confirm these findings.

## Additional Information and Declarations

### Data Availability

The following information was supplied regarding data availability: This is a literature review, and the research in this article did not generate any data or code.
